# Transcribed ultraconserved region Uc.63+ promotes resistance to docetaxel through regulation of androgen receptor signaling in prostate cancer

**DOI:** 10.18632/oncotarget.21688

**Published:** 2017-10-09

**Authors:** Yohei Sekino, Naoya Sakamoto, Keisuke Goto, Ririno Honma, Yoshinori Shigematsu, Kazuhiro Sentani, Naohide Oue, Jun Teishima, Akio Matsubara, Wataru Yasui

**Affiliations:** ^1^ Department of Molecular Pathology, Hiroshima University Institute of Biomedical and Health Sciences, Hiroshima, Japan; ^2^ Department of Urology, Hiroshima University Institute of Biomedical and Health Sciences, Hiroshima, Japan; ^3^ Cancer Biology Program, University of Hawaii Cancer Center, Honolulu, HI, USA

**Keywords:** Uc.63+, prostate cancer, miR-130b, droplet digital PCR, docetaxel

## Abstract

Docetaxel is the standard chemotherapy for metastatic castration-resistant prostate cancer (CRPC). However, nearly all patients ultimately become refractory due to the development of docetaxel resistance. The transcribed ultraconserved regions (T-UCRs) are a novel class of non-coding RNAs that are absolutely conserved across species and are involved in carcinogenesis including prostate cancer (PC). In this study, we investigated the transcriptional levels of 26 representative T-UCRs and determined the regions that were differentially expressed in PC. Quantitative real-time polymerase chain reaction analysis revealed that the expression of T-UCR Uc.63+ was increased in PC tissues. MTT assay and wound healing assay revealed that Uc.63+ was involved in cell growth and cell migration. miR-130b was predicted to have binding sites within the Uc.63+ sequence. The expression of miR-130b was significantly disturbed by the overexpression or knockdown of Uc.63+. We also showed that Uc.63+ regulated the expression of MMP2 via miR-130b regulation. Furthermore, overexpression of Uc.63+ increased the expression of AR and its downstream molecule PSA and promoted resistance to docetaxel through AR regulation. In patients treated with docetaxel, the expression of serum Uc.63+ in the docetaxel-resistant patients was higher than that in the docetaxel-sensitive patients (*P* = 0.011). Moreover, Kaplan-Meier analysis showed that the high expression of serum Uc.63+ correlated with a worse prognosis (*P* = 0.020). These results substantially support the important role that Uc.63+ plays in PC progression by interacting with miR-130b and indicate that Uc.63+ could potentially be a promising serum marker for deciding the best treatment for patients with CRPC.

## INTRODUCTION

Prostate cancer (PC) is the most prevalent cancer among men and the second leading cause of cancer-related death in developed countries [1]. Androgen receptor (AR) is largely involved in the development and growth of PC [2], and most patients with PC are initially sensitive to androgen deprivation therapy. However, most of these patients eventually develop castration-resistant PC (CRPC) (defined as disease progression during androgen-ablation therapy despite secondary hormone therapy) that will inevitably result in metastasis and death [3]. Although docetaxel is the standard chemotherapy for CRPC [4], nearly all patients with docetaxel chemotherapy become refractory probably due to restoration of AR signaling caused by AR gene amplification and mutations, which are critical events in patients with CRPC [5]. Therefore, identifying new molecular mechanisms underlying aberrant AR activation and docetaxel resistance hold great promise to improve chemotherapy for CRPC.

Recently, many reports have shown that noncoding RNAs (ncRNAs) are most likely to be crucial regulators of development and progression in PC [6]. Despite the pivotal role of AR in the development and progression in PC, there is little evidence that ncRNAs have been implicated in the regulation of AR signaling [7], [8]. Among several classes of ncRNAs, a genome-wide survey identified 481 ncRNAs with a size > 200 bp that are absolutely conserved (100%) between the orthologous regions among most of the vertebrate genomes [9], and these have been named transcribed ultraconserved regions (T-UCRs). Given that T-UCRs are conserved across species, they are believed to play critical roles in human development and disease. Two main mechanisms have been described as the regulatory machinery controlling the expression of T-UCRs: interactions with miRNAs and hypermethylation of CpG island promoters [10]. T-UCRs show a ubiquitous or a tissue-specific pattern and also exhibit distinct profiles in various human cancers [11], and whether T-UCRs play an oncogenic role or a tumor-suppressive role depends on the cellular context [11], [12]. Based on this evidence, T-UCRs could provide useful markers to classify the characteristics of human cancer, diagnostic markers for some specific types of cancer, and predictive markers for drug sensitivity or prognosis of cancer patients. We previously showed that some T-UCRs are aberrantly expressed in PC and are silenced by DNA methylation [12]; however, further in-depth studies are needed to determine the complete picture of the biological function of T-UCRs in PC. In this study, we evaluated the expression of T-UCRs in PC, investigated their functional roles in PC progression and AR regulation, and analyzed the effect of Uc.63+ on docetaxel resistance.

## RESULTS

### Expression profiles of T-UCR in PC

**Figure 1:**
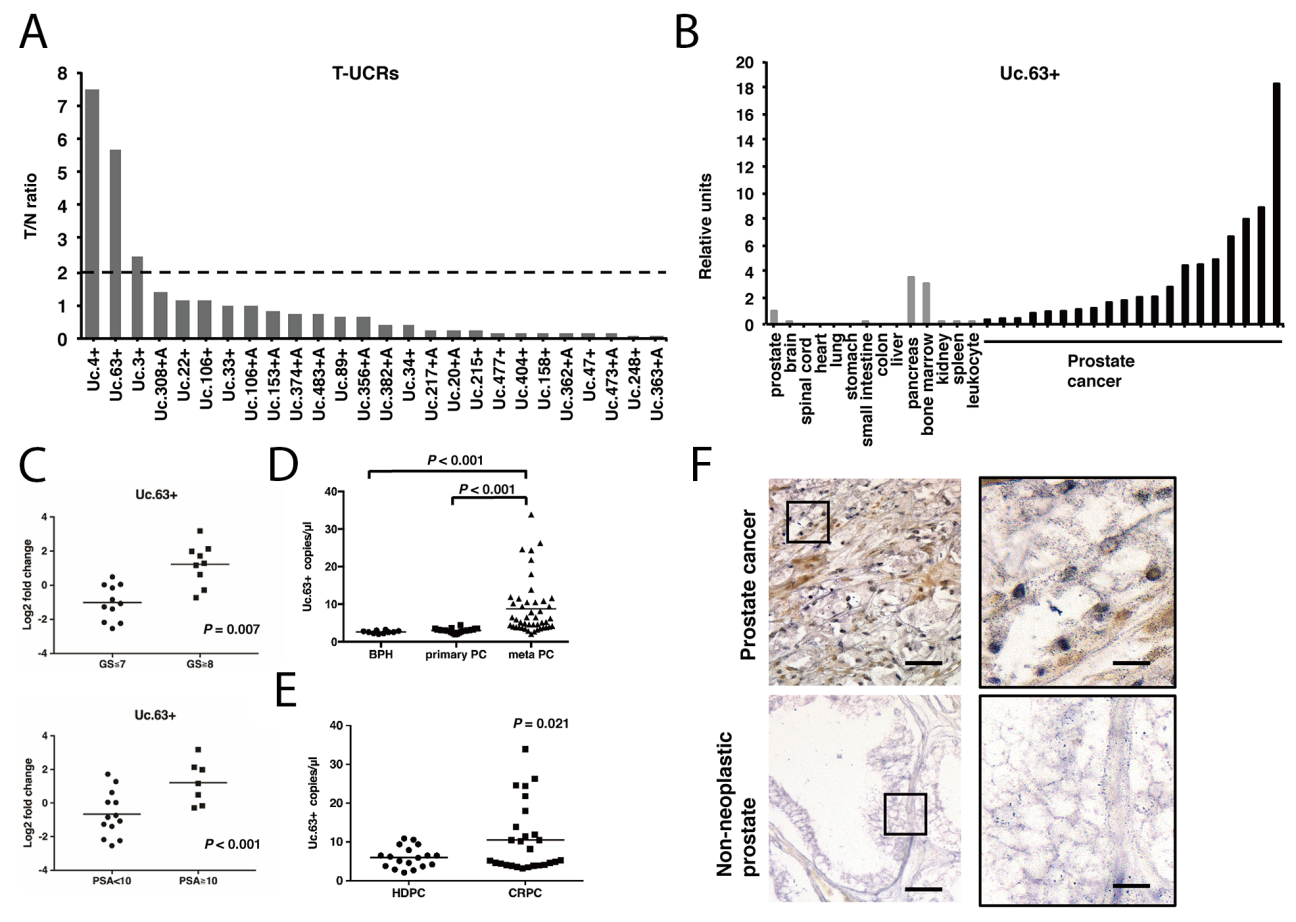
The expression of transcribed ultraconserved regions (T-UCRs) in prostate cancer (PC) **(A)** The fold difference indicates the ratios of T-UCR expression in PC tissues compared with non-neoplastic tissues. **(B)** qRT-PCR analysis of the expression of Uc.63+ in 14 kinds of normal tissues and 20 PC samples. **(C)** Scatter plot diagrams showing the association between the expression of Uc.63+ and clinicopathological findings (Gleason score [GS], prostate-specific antigen [PSA]). Statistical differences were evaluated with the Mann-Whitney *U*-test. **(D)** Results of droplet digital PCR (ddPCR) for the expression of Uc.63+ in serum of patients with benign prostatic hyperplasia (BPH), primary PC (localized PC), and meta PC (PC with metastatic sites). Statistical differences were evaluated with the Mann-Whitney U-test. **(E)** Results of ddPCR for the expression of Uc.63+ in serum in hormone-dependent PC (HDPC) and castration-resistant PC (CRPC). Statistical differences were evaluated with the Mann-Whitney U-test. **(F)** In situ hybridization analysis for the expression of Uc.63+ in PC and non-neoplastic prostate. Representative imagines with low (left panels) and high (right panels) magnifications were shown. Scale bars, 50 μm for low magnification image (left panel); 10 μm for high magnification images (right panels).

To identify ideal biomarkers and therapeutic targets for PC, we first analyzed 26 candidate T-UCRs that were upregulated in PC based on a microarray analysis [13]. To validate the results obtained by a microarray analysis, we examined the expression of these 26 T-UCRs by quantitative real-time polymerase chain reaction (qRT-PCR) using 12 PC tissues and 8 non-neoplastic prostate tissues (Supplementary Figure 1). The mean expression level of each T-UCR was calculated, and the ratio of tumor/non-neoplastic prostate (T/N ratio) was determined (Figure 1A). We identified 3 regions (Uc.3+, Uc.4+, and Uc.63+) that were significantly upregulated in PC tissues compared with non-neoplastic prostate tissues. We then analyzed the expression of these 3 regions in PC cell lines (LNCaP, DU145, and PC3) and revealed that the expression of Uc.63+ in PC cell lines was elevated compared with that in non-neoplastic tissues. In contrast, the expression of Uc.3+ and Uc.4+ in PC cell lines was lower than that in the non-neoplastic prostate tissues (Supplementary Figure 2). Given these results, we decided to focus on Uc.63+. Then, we examined the expression of Uc.63+ in 14 types of normal tissue samples and 20 PC tissues from another cohort/sample set by qRT-PCR. Among these normal tissue samples, the highest expression of Uc.63+ was found in the pancreas. However, the expression of Uc.63+ in PC tissues was higher than that in the pancreas (Figure 1B). In this sample set, the expression of Uc.63+ was significantly associated with high Gleason score (*P* = 0.007) and high prostate-specific antigen (PSA) level (*P* < 0.001) (Figure 1C). To further investigate the usefulness of Uc.63+ as a serum biomarker for PC, we examined the expression of Uc.63+ in the serum of 10 patients with benign prostatic hyperplasia (BPH), 24 patients with primary PC, and 45 patients with metastatic PC by droplet digital PCR (ddPCR). Representative images of ddPCR are shown in Supplementary Figure 3. ddPCR analysis determined that significantly higher expression of Uc.63+ was detected in metastatic PC than in BPH and primary PC (Figure 1D). Moreover, the expression of Uc.63+ was significantly elevated in the serum from primary PC compared with that in the serum from BPH (Supplementary Figure 4). The expression of Uc.63+ was correlated with PSA concentration in the serum from primary PC and metastatic PC. (Supplementary Figure 5). Additionally, we identified significantly higher expression of Uc.63+ in the serum of CRPC patients than that in hormone-dependent PC patients (Figure 1E). To clarify the localization of Uc.63+, we performed in situ hybridization of Uc.63+ in PC tissues and found that robust expression of Uc.63+ was observed in the nucleus in PC tissues, whereas the expression of Uc.63+ was rarely detected in non-neoplastic prostate tissues (Figure 1F).

### Uc.63+ acts as an oncogene and is associated with cell proliferation and cell migration

**Figure 2:**
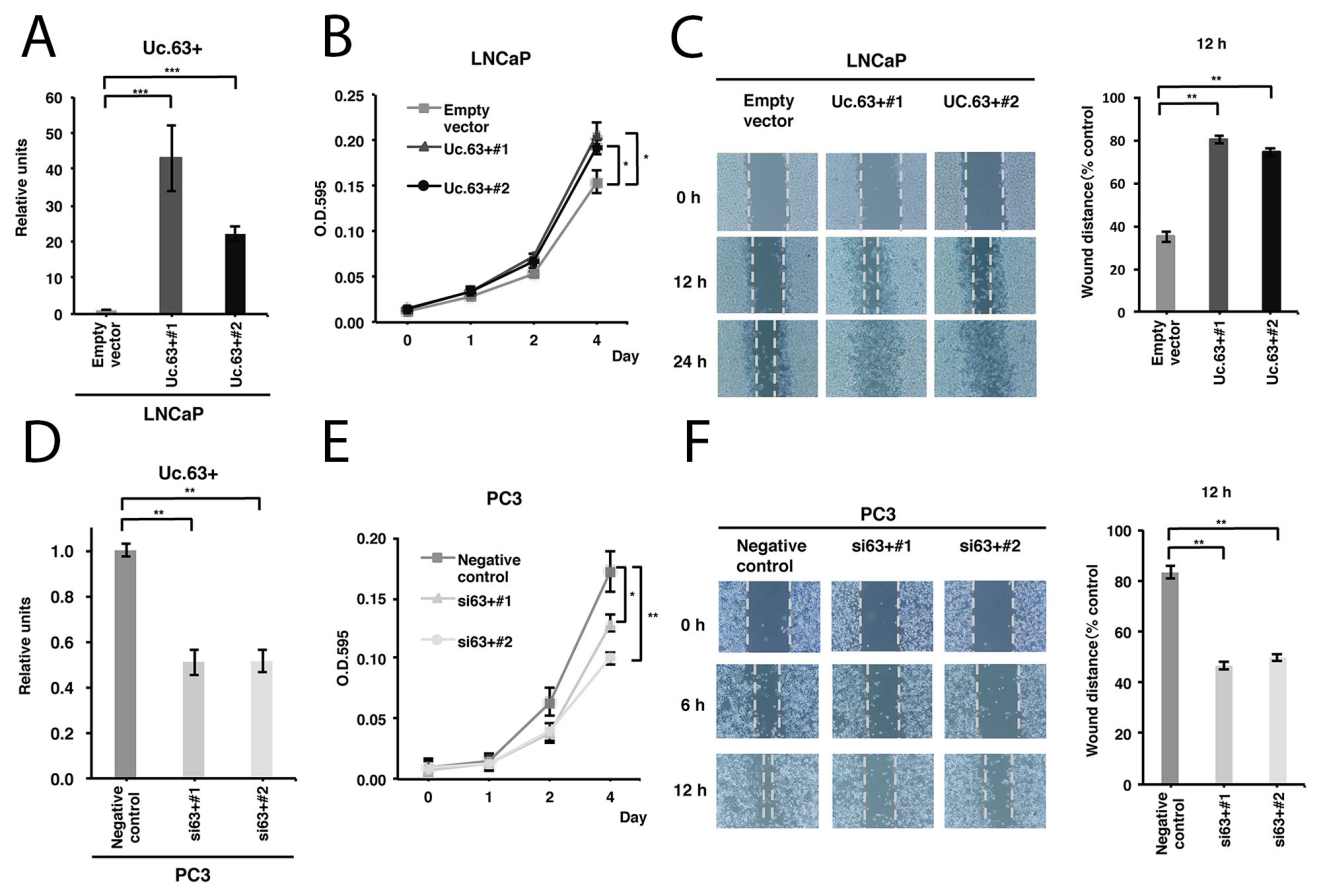
Uc.63+ promotes cell proliferation and migration in prostate cancer (PC) cells **(A)** The result of qRT-PCR for the expression of Uc.63+ in LNCaP cells transfected with Uc.63+ expression vector or empty vector. The results are expressed as the mean ± S.D. of triplicate measurements. ^***^P <0.001. **(B)** Cell proliferation assay in LNCaP cells transfected with the Uc.63+ expression vector or empty vector. Cell growth was assessed by MTT assays at 1, 2, and 4 days after seeding on 96-well plates. Bars and error bars are the mean and S.D., respectively, of 3 independent experiments. ^*^P <0.05. **(C)** Representative images of a wound healing assay in LNCaP cells transfected with the Uc.63+ expression vector or empty vector. Wound closures were evaluated by wound contraction percentage and closure time at 0, 12, and 24 hours after scratching. The results are expressed as the mean and S.D. of triplicate measurements. ^**^P <0.01. **(D)** The results of qRT-PCR for the expression of Uc.63+ in PC3 cells transfected with negative control or two different siRNAs. The results are expressed as the mean and S.D. of triplicate measurements. ^**^P <0.01. **(E)** Cell proliferation assay in PC3 cells transfected with negative control or two different siRNAs. Cell growth was assessed by MTT assays at 1, 2, and 4 days after seeding on 96-well plates. Bars and error bars are the mean and S.D., respectively, of 3 independent experiments. ^*^P <0.05, ^**^P <0.01. **(F)** Representative images of wound healing assays in PC3 cells transfected with negative control or two different siRNAs. Wound closures were evaluated by wound contraction percentage and closure time at 0, 6, and 12 hours after scratching. The results are expressed as the mean and S.D. of triplicate measurements. ^**^P <0.01.

To elucidate the biological roles of Uc.63 in PC, we first constructed a vector containing the whole sequence of Uc.63+ and transfected it into LNCaP cells that possessed a low level of Uc.63+. As shown in Figure 2A, transfection of the vector significantly induced Uc.63+ overexpression. Next, we performed a 4,5-dimethylthiazol-2-yl-2,5-diphenyltetrazolium bromide (MTT) assay and wound healing assay. LNCaP cells transfected with the Uc.63+ expression vector showed significantly increased cell proliferation and migration (Figure 2B, 2C). To further verify whether Uc.63+ is required for cell proliferation and migration, we investigated the effects on the downregulation of Uc.63+ by using small interfering RNA (siRNA) that was designed to specifically target Uc.63+. We confirmed a significantly lower expression of Uc.63+ in DU145 and PC3 cells with two different siRNAs than that in the negative control (Figure 2D). Following these results, we examined the MTT assay and wound healing assay and found that knockdown of Uc.63+ reduced cell proliferation and migration (Figure 2E, 2F). These results showed that Uc.63+ has an apparent oncogenic role in PC.

### Uc.63+ and XPO1 are independently regulated

Based on data from the National Center for Biotechnology Information, Uc.63+ is located in the third intron of the *XPO1* gene on chromosome 2 (Supplementary Figure 6A). To evaluate the relationship between Uc.63+ and the host gene *XPO1* transcription, we examined the expression of *XPO1* by qRT-PCR in PC tissues. We designed the primers to cover one of the exons of *XPO1*, which does not include the Uc.63+ coding region. The expression of *XPO1* did not correlate with that of Uc.63+ (Supplementary Figure 6B). As we expected, knockdown of the expression of Uc.63+ had no effect on the expression of *XPO1* in DU145 and PC3 cells, and the expression of *XPO1* was not changed in LNCaP cells transfected with the Uc.63+ expression vector (Supplementary Figure 6C, 6D). These results suggested that the expression of Uc.63+ and *XPO1* are independently regulated, which is consistent with a previous finding [14].

### Uc.63+ on its own enhances the expression of AR and its downstream gene *PSA*

**Figure 3:**
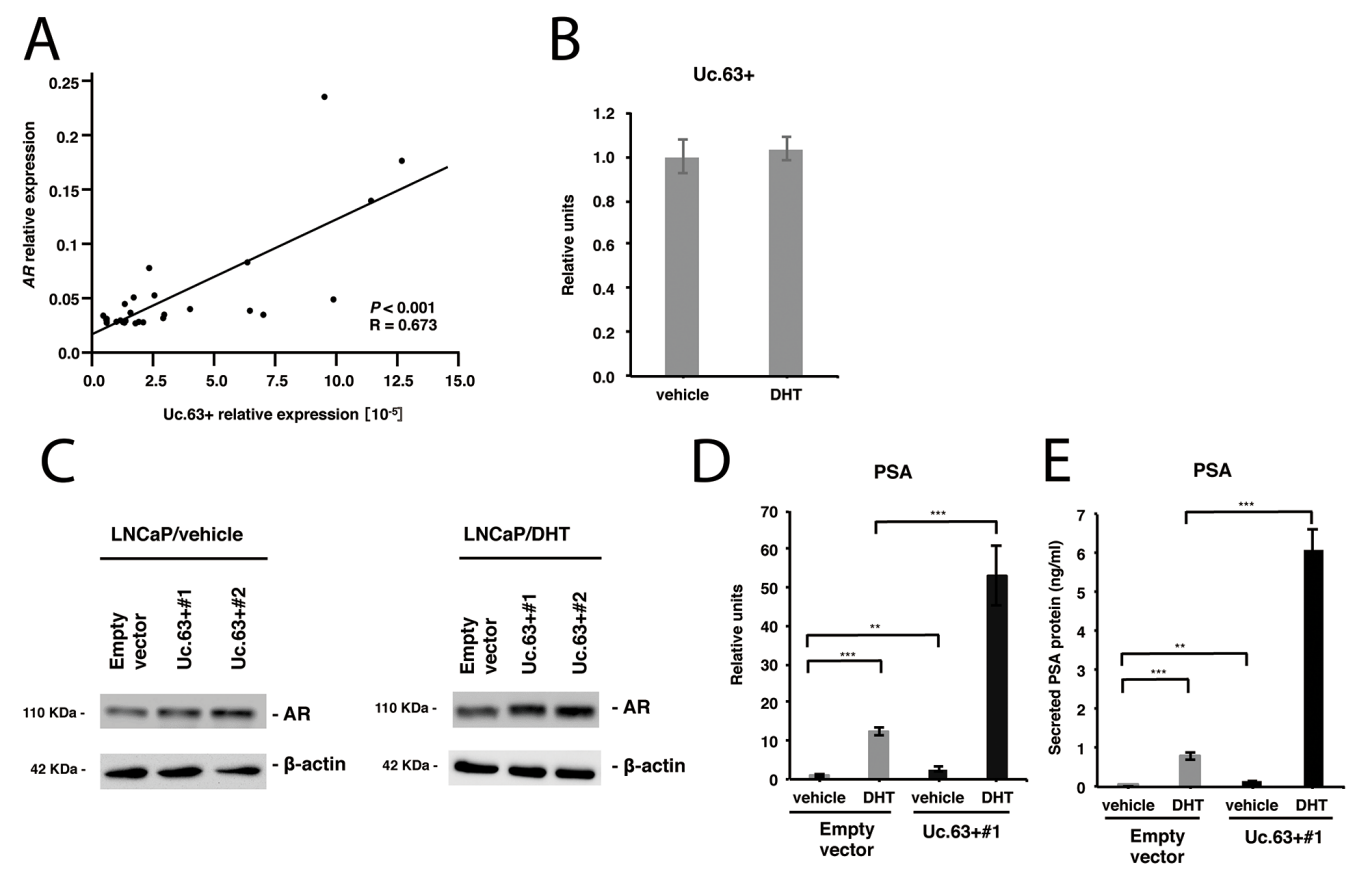
Uc.63+ modulates androgen receptor (AR) and its downstream gene *PSA* **(A)** The correlation between the expression of Uc.63+ and the expression of *AR* in prostate cancer (PC) tissues. Spearman correlation coefficient and *P*-values are indicated. **(B)** qRT-PCR analysis for the expression of Uc.63+ in LNCaP cells in the presence of dihydrotestosterone (DHT) (10 nM) or vehicle (ethanol). **(C)** Western blot analysis of AR in LNCaP cells transfected with Uc.63+ expression vector or empty vector in the presence of DHT (10 nM) or vehicle (ethanol). β-actin was used as a loading control. **(D, E)** mRNA expression of *PSA* and secreted protein expression of PSA in LNCaP cells transfected with Uc.63+ expression vector or empty vector in the presence of DHT (10 nM) or vehicle (ethanol). The results are expressed as the mean and S.D. of triplicate measurements. ^**^P <0.01, ^***^P <0.001.

AR plays a central role in PC carcinogenesis, and a number of regulators of the AR signaling pathway have been identified [6]. To investigate whether Uc.63+ is involved in this pathway, we examined the expression of *AR* in PC tissues and non-neoplastic prostate tissues by qRT-PCR and found that the expression of Uc.63+ correlated positively with that of AR (Figure 3A). Then, we observed the expression of Uc.63+ in LNCaP cells after treatment with dihydrotestosterone (DHT) or vehicle to verify whether androgen regulated the expression of Uc.63+. Treatment with both DHT and with vehicle did not yield any significant upregulation of the expression of Uc.63+ (Figure 3B), which suggested that the expression of Uc.63+ is regulated in an androgen-independent manner. To investigate a possible role of Uc.63+ in the AR signaling pathway, we examined the expression of AR in LNCaP cells that are well established as an AR-positive PC cell line. Western blot analysis revealed that overexpression of Uc.63+ increased the expression of AR in the presence or absence of DHT (Figure 3C). Additionally, we investigated the ability of Uc.63+ to regulate the AR downstream gene *PSA*. The expression of *PSA* was clearly induced in LNCaP cells treated with DHT compared to that in LNCaP cells treated with vehicle. Overexpression of Uc.63+ also increased the expression of *PSA* in the presence or absence of DHT (Figure 3D). Similar results were obtained at the secreted protein PSA level (Figure 3E). Knockdown of Uc.63+ did not affect the expression of AR and *PSA* in DU145 cells, which were shown to be AR-negative cells by western blot analysis (Supplementary Figure 7A, 7B).

### Uc.63+ promotes docetaxel resistance in CRPC patients

**Figure 4:**
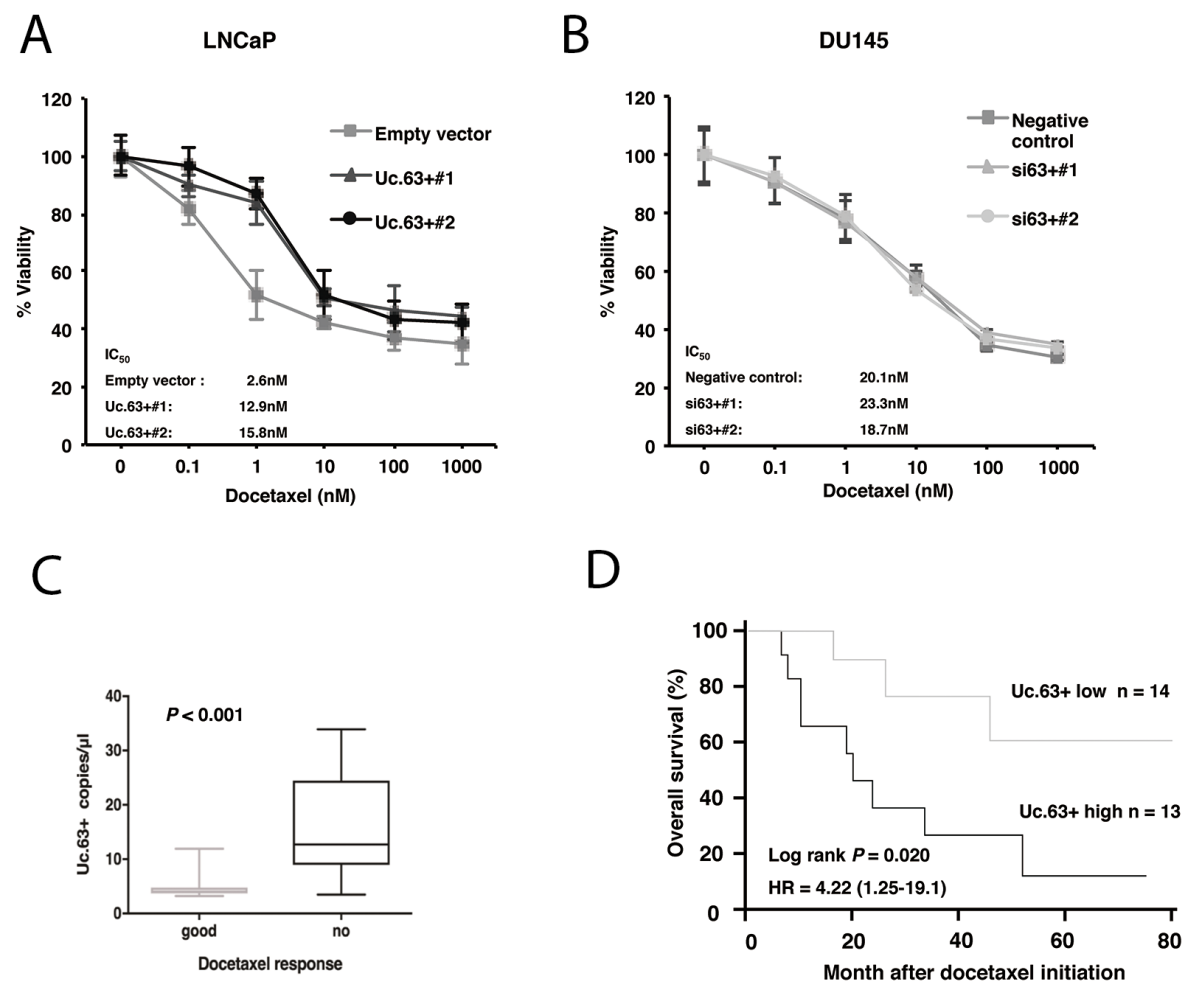
Effect of Uc.63+ on docetaxel sensitivity in prostate cancer (PC) cell lines and on clinical outcome **(A)** Dose-dependent effect of docetaxel on the viability of LNCaP cells transfected with Uc.63+ expression vector or empty vector. **(B)** Dose-dependent effect of docetaxel on the viability of DU145 transfected with negative control or two different siRNAs. **(C)** Box-plot representing the expression of Uc.63+ in chemo-sensitive and chemo-resistant patients with castration-resistant prostate cancer (CRPC). **(D)** Kaplan-Meier curves for overall survival of patients with CRPC and docetaxel chemotherapy.

Several studies showed that there could be an interaction between AR signaling activity and docetaxel sensitivity [15]. Therefore, we examined the involvement of Uc.63+ in docetaxel resistance. We performed MTT assays to measure cell viability in LNCaP cells transfected with Uc.63+ expression vector and empty vector under various concentrations of docetaxel for 48 hours in the absence of DHT. The IC_50_ value of LNCaP cells transfected with Uc.63+ expression vector was significantly higher than that of LNCaP cells transfected with empty vector (Figure 4A). To further address whether the observed effect of Uc.63+ on docetaxel resistance is specifically caused by AR upregulation, we used DU145 cells, which are well known as an AR-negative cell line. As shown in Figure 4B, knockdown of Uc.63+ had no effect on docetaxel sensitivity in the DU145 cells. To rule out the possibility that miR-130b was involved in docetaxel resistance, we examined the effect of miR-130b on docetaxel resistance. Overexpression of miR-130b did not affect docetaxel sensitivity in LNCaP cells transfected with Uc.63+ (LNCaP Uc.63+#1) (Supplementary Figure 8). Hence, we concluded that the effect of Uc.63+ on docetaxel resistance is mediated mainly by regulation of AR. We then elucidated the utility of Uc.63+ as a serum marker for predicting therapeutic outcomes of PC patients. We performed ddPCR in 27 patients with metastatic CRPC who were treated with docetaxel chemotherapy. We selected a cut-off point at the median serum level of Uc.63+ in the ddPCR data. The patients with high expression of UC.63+ had a lower PSA response rate than the patients with low expression of Uc.63+ (*P* = 0.078) (Supplementary Figure 9). Although these differences did not quite reach statistical significance, Uc.63+ serum levels were significantly higher in the docetaxel-resistant patients than in the docetaxel-sensitive patients (*P* = 0.011) (Figure 4C). Moreover, Kaplan-Meier analysis showed the expression of Uc.63+ to be significantly associated with poor therapeutic outcomes in patients with metastatic CRPC (Log rank test, *P* = 0.02; hazard ratio 4.2) (Figure 4D).

### Interaction between Uc.63+ and miR-130b

**Figure 5:**
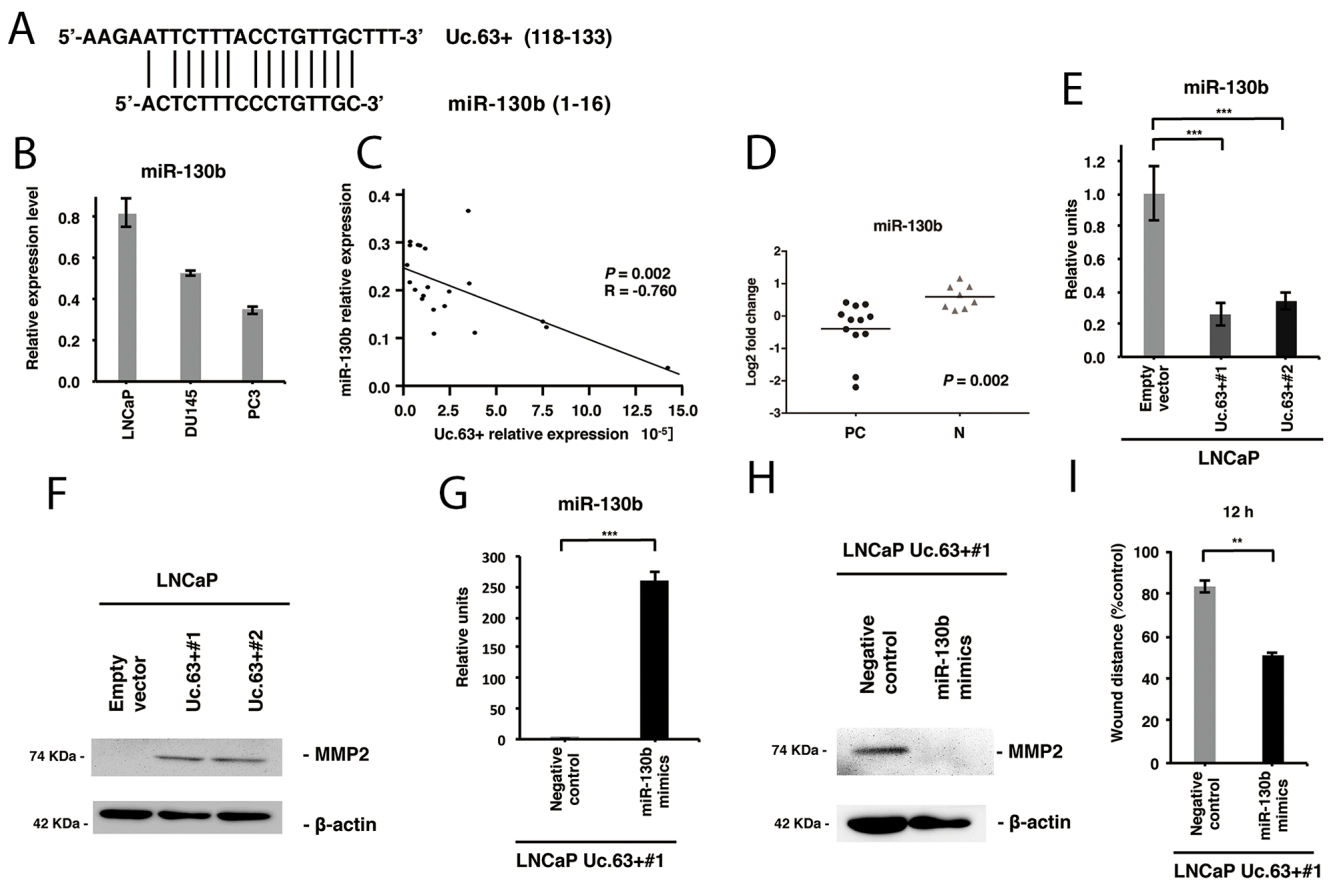
Uc.63+ increases MMP2 through regulation of miR-130b **(A)** The complementary sites between Uc.63+ and miR-130b. **(B)** The result of qRT-PCR for the expression of miR-130b in prostate cancer (PC) cell lines (LNCaP, DU145, and PC3). **(C)** The correlation between Uc.63+ and miR-130b in PC tissues. Spearman correlation coefficient and *P*-values are indicated. **(D)** The result of qRT-PCR for the expression of miR-130b in PC tissues and non-neoplastic prostate (N) tissues. **(E)** qRT-PCR analysis for the expression of miR-130b in LNCaP cells transfected with Uc.63+ expression vector or empty vector. The results are expressed as the mean and S.D. of triplicate measurements. ^***^P <0.001. **(F)** Western blot analysis of MMP2 and β-actin in LNCaP cells transfected with Uc.63+ expression vector or empty vector. β-actin was used as a loading control. **(G)** The result of qRT-PCR for the expression of miR-130b in LNCaP cells transfected with Uc.63+ (LNCaP Uc.63+#1) transfected with miR-130b mimics or negative control. The results are expressed as the mean and S.D. of triplicate measurements. ^***^P <0.001. **(H)** Western blot analysis of MMP2 and β-actin in LNCaP Uc.63+#1 cells transfected with miR-130b mimics or negative control. β-actin was used as a loading control. **(I)** Wound healing assay in LNCaP Uc.63+#1 cells transfected with miR-130b mimics or negative control. Wound closures were evaluated by wound contraction percentage and closure time at 0, 12, and 24 hours after scratching. The results are expressed as the mean and S.D. of triplicate measurements. ^**^P <0.01.

Recent evidence suggests that T-UCRs act as endogenous competing RNAs, and miRNA is one of the key regulators of T-UCRs [16]. To identify the miRNAs that could potentially interact with Uc.63+, we used two online software programs: UCbase 2.0 and miRanda. We detected that miR-130b and miR-605 had complementary sequences of Uc.63+ (Figure 5A). Further validation study using qRT-PCR disclosed that the expression of miR-130b was robust both in PC tissues and in non-neoplastic prostate tissues, whereas miR-605 was not detectable in either of them (data not shown). Therefore, we focused on miR-130b. A higher expression of miR-130b in LNCaP cells was detected in comparison with that in DU145 or PC3 cells (Figure 5B). Furthermore, we investigated the expression of miR-130b in PC tissues and non-neoplastic prostate tissues by qRT-PCR and found that the expression of miR-130b inversely and significantly correlated with the expression of Uc.63+ (*P* = 0.002) (Figure 5C). There was significant downregulation of miR-130b in PC tissues compared with non-neoplastic prostate tissues (*P* = 0.002) (Figure 5D). To further investigate the interaction between Uc.63+ and miR-130b, we examined the effect of Uc.63+ deregulation on the expression of miR-130b. Overexpression of Uc.63+ in LNCaP cells downregulated miR-130b (Figure 5E). Meanwhile, silencing of Uc.63+ in PC3 cells upregulated the expression of miR-130b (Supplementary Figure 10A). It has been shown that matrix metallopeptidase 2 (MMP2) is a direct target of miR-130b [17]. Western blot analysis revealed that MMP2 was higher in LNCaP cells transfected with Uc.63+ expression vector than that in LNCaP cells transfected with empty vector (Figure 5F). As we expected, silencing of Uc.63+ in PC3 cells downregulated the expression of MMP2 by western blot analysis (Supplementary Figure 10B). To further verify that Uc.63+ was involved in the oncogenic role through miR-130b, we examined the overexpression of miR-130b in LNCaP Uc.63+#1 cells. qRT-PCR showed the expression of miR-130b was higher in LNCaP Uc.63+#1 cells transfected with miR-130b mimics than that in LNCaP Uc.63+#1 cells transfected with negative control (Figure 5G). Overexpression of miR-130b in LNCaP Uc.63+#1 cells downregulated the expression of MMP2 and migratory ability (Figure 5H, 5I). These results suggest that Uc.63+ may upregulate the expression of MMP2 through miR-130b interaction, which is most likely to contribute to the oncogenic role of Uc.63+.

## DISCUSSION

As we have already mentioned, T-UCRs are absolutely conserved among most of the vertebrate genomes, which means that T-UCRs could potentially play essential roles in ubiquitous vital phenomenon in comparison with the other classes of non-coding RNAs. Prior studies have emphasized that there are distinct signatures related to the expression of T-UCRs in human cancers, and some of the T-UCRs supposedly have functional roles in cancer biology, which implied that T-UCRs could provide valuable molecular markers and/or therapeutic targets. In the work presented here, we identified the signature of T-UCRs that were differentially expressed in PC and extensively studied T-UCR Uc.63+ as it was significantly upregulated in both PC tissues and cell lines. One recent study reported a T-UCR signature that was overexpressed in PC tissue through a microarray analysis using 57 PC and 7 non-neoplastic prostate tissues; however, Uc.63+ was not chosen as a hopeful candidate. The actual strategy we applied in this study was to validate the expression of 26 T-UCRs with qRT-PCR by referring to the signatures reported by Hudson et al [13], which enabled us to focus on Uc.63+, and its functional roles in PC were quite consistent with those determined in a previous study on breast cancer [14]. One of the plausible explanations for the oncogenic role of Uc.63+ is thought to be an interaction with miR-130b that regulates MMP2. Several lines of study have provided evidence for an important role of the interaction between T-UCRs and miRNA in cancer biology. In this present study, we focused only on the interaction between Uc.63+ and miR-130b, which would surely explain how Uc.63+ is involved in the regulation of PC cell migration and invasion but is not accountable enough for the interaction among Uc.63+ and PC cell growth. There could probably be a bunch of unknown miRNAs that are potentially regulated by Uc.63+, which may help to explain how Uc.63+ contributes to PC cell growth activity. These findings call for more studies on the interaction between T-UCRs and miRNAs to elucidate a fuller picture of the molecular mechanism in cancer biology.

We found that the expression of Uc.63+ correlated significantly with that of AR *in vivo* and that Uc.63+ could directly regulate the expression of AR in an androgen-independent manner *in vitro*, which led to docetaxel resistance in PC cells. To our knowledge, this is the first study to report the direct effect of T-UCRs on AR signaling and its downstream target PSA and the involvement of T-UCRs in the drug resistance of cancer cells. Several lines of evidence have shown that AR signaling modulates docetaxel sensitivity [15], [18], [19], and the recent CHAARTED clinical trial showed that patients with hormone-sensitive metastatic PC who received docetaxel chemotherapy given at the time of androgen deprivation therapy lived more than one year longer than patients who received androgen deprivation therapy alone [20]. We showed that overexpression of Uc.63+ enhanced docetaxel resistance in androgen-dependent LNCaP cells, and knockdown of Uc.63+ had no effect on the docetaxel resistance in androgen-independent DU145 cells. These results further support the notion that Uc.63+ is surely involved in modulation of the docetaxel sensitivity of PC cells through regulation of AR signaling. Docetaxel is the only well-established chemotherapeutic drug that provides a significant benefit to clinical outcome in CRPC patients [4]. To fully determine the machinery that controls docetaxel resistance in PC cells, further in-depth studies on which molecule in AR signaling is actually regulated by UC.63+ need to be elucidated; this could be a potential game changer in the treatment of PC.

One of the most important findings of this study was that we successfully detected the expression of Uc.63+ in serum samples from PC patients using ddPCR, which is believed to be a much more sophisticated method than conventional qRT-PCR. Although several studies have reported the expression of some T-UCRs in cancer tissues and cancer cell lines [21] [12], there are no reports of the detection of T-UCRs in serum samples from cancer patients. We provided the first evidence that T-UCRs can be detected in serum samples from cancer patients, suggesting that T-UCRs are more likely to be promising serum markers. Although PSA has been mainly used as a serum marker for PC, one of the issues with PSA is its low specificity in PC patients and false-positive results in BPH patients [22]. In contrast, the expression of Uc.63+ was significantly upregulated in the serum from PC patients compared with that from BPH patients. Moreover, we revealed that the expression of Uc.63+ in serum was remarkably upregulated in CRPC patients, and evaluation of its expression level in PC patients could be a useful biomarker for predicting the sensitivity to docetaxel treatment and clinical outcome for patients that receive docetaxel treatment. To date, we have no clinical applications to predict the response to docetaxel chemotherapy in PC patients. The present results suggested that monitoring the expression of Uc.63+ in serum could potentially improve several steps of PC treatment, including the screening of PC patients using serum samples, and contribute to selection of the best treatment for PC patients: so-called “tailor-made treatment”. However, one concern is that the robust expression of Uc.63+ was observed in the nucleus in PC tissues, which indicates to us that the mechanism behind the secretion of nuclear Uc.63+ into serum is unclear. A recent report showed that the localization of Uc.8 was changed from the nucleus to the cytoplasm according to the tumor grading [23]. It is our current important task to determine the fundamental machinery of Uc.63+ secretion, which could further support the utility of Uc.63+ as a serum biomarker in PC patients. What is more, although expression and function of AR is still relevant in CRPC, we used DU145 and PC3 (AR negative cells) as CRPC model in this research. CRPC cell lines which express AR will be needed to verify the current data. In closing, we found that T-UCR Uc.63+ was overexpressed in PC and showed that Uc.63+ modulated the expression of MMP2 via the regulation of miR-130b. Additionally, Uc.63+ promoted docetaxel resistance by regulating the expression of AR. Furthermore, the expression of Uc.63+ in the serum was dramatically changed through the carcinogenesis and progression of PC from BPH to CRPC. Although additional studies will likely be needed to further advance our understanding of how Uc.63+ contributes to PC progression, the data presented here highlight the great potential of Uc.63+ as a serum biomarker and therapeutic target in patients with PC.

## MATERIALS AND METHODS

### Cell lines

Three PC cell lines (LNCaP, PC3, and DU145) were purchased from the American Type Culture Collection (Manassas, VA, USA). PC3 and DU145 cell lines were maintained in RPMI 1640 (Nissui Pharmaceutical Co. Ltd., Tokyo, Japan) containing 10% fetal bovine serum (FBS, BioWhittaker, Walkersville, MD, USA), 2 mM L-glutamine, 50 U/mL penicillin, and 50 g/mL streptomycin in a humidified atmosphere of 5% CO_2_ and 95% air at 37°C. LNCaP cells were withdrawn from hormone effects in the medium by culture with medium containing charcoal/dextran stripped FBS for 2 days before treatment. DHT (10 nM) or vehicle control (0.5% ethanol) was added to the cells. Some experiments were examined 24 h later.

### Tissue samples

For qRT-PCR, 20 PC tissue samples and 8 non-neoplastic prostate tissue samples were used (Supplementary Table 1). The non-neoplastic prostate tissue samples were obtained at biopsy or autopsy, as described as previously [24]. The samples were immediately frozen in liquid nitrogen and stored at -80°C until use. For ddPCR, 5-mL serum samples collected from 79 patients, including 10 with BPH, 24 with localized PC, 18 with hormone-dependent PC, and 27 with CRPC, were used (Supplementary Table 2). The Institutional Review Board of Hiroshima University Hospital approved this study (approval no.E-688), and appropriate informed consent was obtained from each patient. The samples were collected from patients at Hiroshima University Hospital or an affiliated hospital. This study was conducted in accordance with the Ethical Guidance for Human Genome/Gene Research of the Japanese Government.

### Quantitative RT-PCR analysis

Total RNA was isolated from frozen samples or cancer cell lines using Isogen (Nippon Gene, Tokyo, Japan), and 1 g of total RNA was converted to cDNA with a First Strand cDNA Synthesis Kit (Amersham Biosciences Corp., Piscataway, NJ, USA). The qPCR was performed with a SYBR Select Master Mix (Applied Biosystems, Austin, TX, USA). The primer sequences for the detection of each T-UCR, *AR*, *PSA*, or *XPO1* are described in Supplementary Table 3. Real-time detection of the emission intensity of SYBR green bound to double-stranded DNA was performed with a CFX Connect Real-Time System (Bio-Rad Laboratories, Hercules, CA, USA). *ACTB*-specific PCR products, which were amplified from the same RNA samples, served as internal controls. To quantify the level of miRNAs, TaqMan assays were performed as described previously [25] The expression values were normalized to the expression of the small RNA gene RNU6, and the relative quantification was determined using the ΔΔCt method as described previously [26].

### Droplet digital PCR

Primers and TaqMan probes are described in Supplementary Table 4. ddPCR samples for Uc.63+ were prepared with a 20-μL reaction mixture containing 10 μL ddPCR Supermix for Probes (no dUTP, Bio-Rad), 500 nM of each forward primer and reverse primer, and 250 nM probe (FAM), and synthesized cDNA droplets were generated with 70 μL of oil using a QX100 droplet generator (Bio-Rad). Amplification was performed at 95°C for 10 min, followed by 40 cycles at 94°C for 30 s and 40 cycles at 57°C for 1 min using a C1000 Touch thermocycler (Bio-Rad). After amplification, the droplets were read on a QX100 droplet reader (Bio-Rad) and analyzed with QuantaSoft software V1.7.4 (Bio-Rad). The QuantaSoft software measured the number of positive versus negative droplets. Their ratio was then fitted to a Poisson distribution to determine the copy number of the target molecule as copies/μL.

### In situ hybridization

Sections 10-μm thick were rehydrated as described above. Thereafter, sections were treated with 0.2 M sodium chloride and proteinase K. Slides were post-fixed, and sections were then demethylated with acetic anhydride and prehybridized. Hybridization was carried out in a humid chamber with 500 ng/mL of freshly prepared digoxigenin-labelled RNA probe of *UC63*. Sections were incubated for at least 48 hours at 65°C. The slides were washed and incubation of the secondary anti-digoxigenin antibody (Roche Diagnostics, Indianapolis, IN, USA) was carried out at 4°C overnight. The next day, sections were washed and developed using Nitro Blue Tetrazolium Chloride/5-Brom-4-Chlor-3-Indolyl-Phosphat (Roche Diagnostics, Indianapolis, IN, USA).

### RNA interference (RNAi) and expression vector

Silencer^®^ Select (Ambion, Austin, TX) against Uc.63+ was used for RNA interference. Two independent oligonucleotides and negative control siRNA (Invitrogen, Carlsbad, CA, USA) were used. The sequence of siRNA#1 was 5’-AAAGAUGUUAACACUACCUga-3’, and that of siRNA#2 was 5’-UUUGGUGCUAAAUUUAUGCac-3’. A total of 1.0 x 10^6^ cells of PC3 or DU145 were plated on a 10-cm culture dish 24 h before transfection. Transfection was performed using Lipofectamine RNAiMAX (Invitrogen, Carlsbad, CA, USA) according to the manufacturer’s instructions. Cells were used 48 h after transfection in each of the experiments and assays. For constitutive expression of Uc.63+, cDNA was PCR amplified and subcloned into pcDNA 3.1 (Invitrogen, Carlsbad, CA, USA). The pcDNA-Uc.63+ expression vector was transfected into LNCaP cells with FuGENE6 (Roche Diagnostics), according to the manufacturer’s instructions.

### Cell growth assay and wound healing assay

To examine cell growth, an MTT assay was performed as described previously [27]. Cell growth was monitored after 1, 2, and 4 days. Cells were seeded onto fibronectin-coated 6-well plates in 2 mL medium and incubated at 37°C for 12 h. A clear area was scraped with a plastic tip. Migration of cells into wounded areas was evaluated with an inverted microscope [28].

### Western blot analysis

Cells were lysed in SDS buffer. Concentrations were determined by Bradford protein assay (Bio-Rad, Richmond, CA, USA) with BSA used as the standard. The lysates (40 μg) were solubilized in Laemmli sample buffer by boiling and then separated by 10% sodium dodecyl sulfate polyacrylamide gel electrophoresis followed by electro-transfer onto a nitrocellulose filter. The filter was incubated for 1 h at room temperature with primary antibody. AR antibody (Thermo Fisher Scientific, MA, USA) and MMP2 antibody (Kyowa Pharma Chemical, Toyama, Japan) were used. Peroxidase-conjugated anti-mouse or anti-rabbit IgG was used in the secondary reaction. Immunocomplexes were visualized with an ECL Plus Western Blot Detection System (Amersham Biosciences, Piscataway, NJ, USA). β-Actin (Sigma-Aldrich, MO, USA) was detected as a loading control.

### Secreted PSA protein level in cell culture supernatants

Secreted PSA protein levels in cell culture supernatants were determined by LUMIPULSE Presto PSA (Fujirebio Inc., Tokyo, Japan) according to the manufacturer’s instructions. Serial dilutions of recombinant human PSA were used to plot the standard curve.

### Drug treatment

Docetaxel was obtained from Sanofi-Aventis and handled according to the manufacturer’s recommendations. Cell lines treated with vehicle (0.5% ethanol) or escalating doses of docetaxel were assessed for cell viability. MTT assay was performed at 48 hours after docetaxel chemotherapy [29]. Drug sensitivity curves and IC_50_ values were calculated using GraphPad Prism 4.0 software (GraphPad Software Inc., San Diego, CA, USA). The main assessment of the end-point of chemotherapy was PSA response according to the criteria of the Prostate Cancer Clinical Trials Working Group (PCWG3) [30]. The PCWG3 defines progressive disease as an increase of ≥25% (at least 2 ng/mL) over the baseline value after 12 weeks of chemotherapy, with confirmation by a second PSA value at least 3 weeks later. Treatment outcome of these 27 patients are summarized in Supplementary Table 5.

### Transfection of miR-130b mimics

miR-130b mimics (target sequence ACUCUUUCCCUGUUGCACUAC, Thermo Fisher Scientific, MA, USA) was used for transfection of miR-130b. Transfection was performed with Lipofectamine RNAiMAX (Invitrogen, Carlsbad, CA, USA). Following the manufacture’s protocol.

### Statistical analysis

All experiments were repeated at least three times with each sample in triplicate. The results are expressed as the mean ± S.D. of triplicate measurements. Sample sizes for relevant experiments were determined by power analysis. Statistical differences were evaluated using the two-tailed Student *t*-test or Mann-Whitney *U*-test. Statistical analyses were conducted primarily using GraphPad Prism software (GraphPad Software Inc.).

## SUPPLEMENTARY MATERIALS FIGURES AND TABLES





## Abbreviations

MTT: 4,5-dimethylthiazol-2-yl-2,5-diphenyltetrazolium bromide
AR: Androgen receptor
BPH: Benign prostatic hyperplasia
CRPC: Castration-resistant prostate cancer
DHT: Dihydrotestosterone
ddPCR: Droplet digital PCR
HDPC: Hormone-dependent PC
ncRNAs: Noncoding RNAs
MMP2: Matrix metallopeptidase 2
meta PC: Metastatic PC
PC: Prostate cancer
PCWG3: Prostate Cancer Clinical Trials Working Group
PSA: Prostate-specific antigen
T/N ratio: Ratio of tumor/non-neoplastic prostate
T-UCRs: Transcribed ultraconserved regions
