# Supplementary material for: Transcribed ultraconserved region Uc.63+ promotes resistance to docetaxel through regulation of androgen receptor signaling in prostate cancer
Source: Oncotarget. 2017 Oct 9;8(55):94259–70. doi: 10.18632/oncotarget.21688 (PMC5706872; doi:10.18632/oncotarget.21688)
Supplement: Supplementary file 1 [file oncotarget-08-94259-s001.pdf]

Transcribed ultraconserved region Uc.63+ promotes resistance to docetaxel through regulation of androgen receptor signaling in prostate cancer

SUPPLEMENTARY MATERIALS

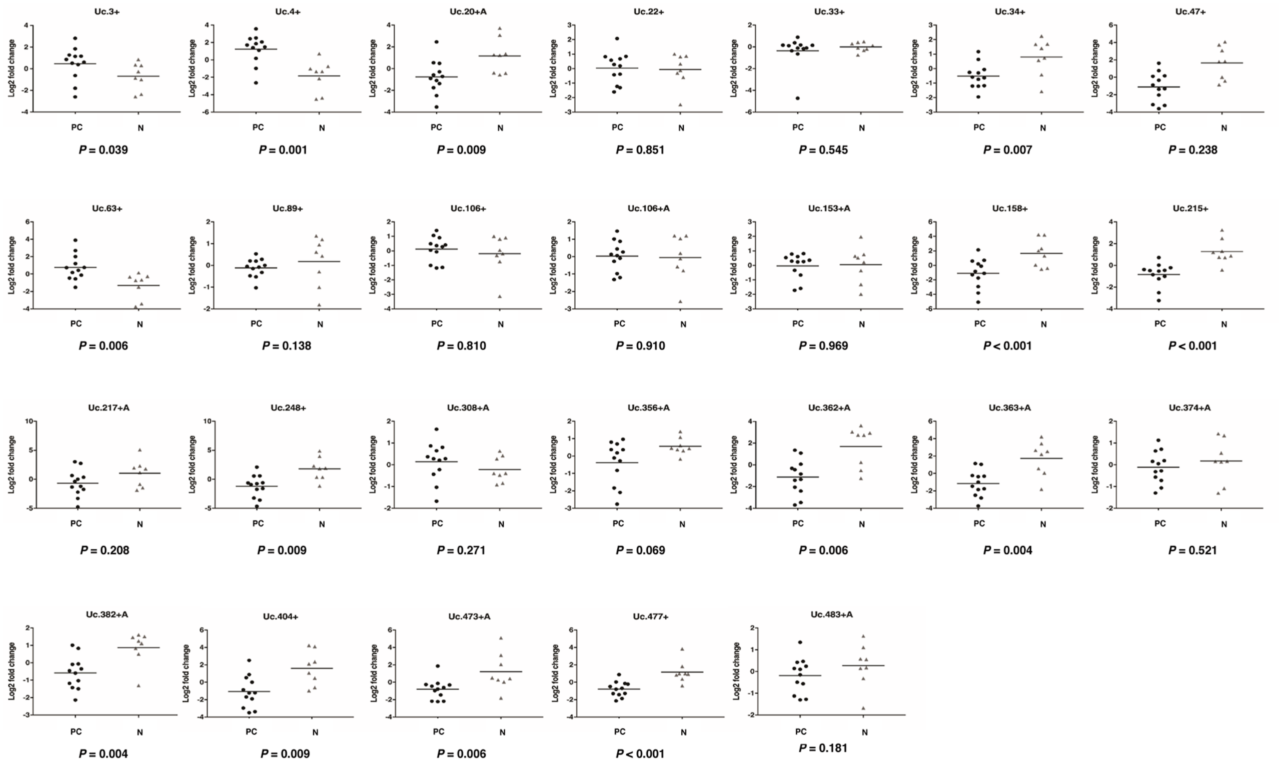

Supplementary Figure 1: The expression of transcribed-ultraconserved regions (T-UCRs) in prostate cancer. Graphs show the results of qRT-PCR for the 26 representative T-UCRs in prostate cancer tissues compared to non-neoplastic prostate tissues. Statistical differences were evaluated with the Mann-Whitney *U*-test. PC, prostate cancer; N, non-neoplastic prostate.

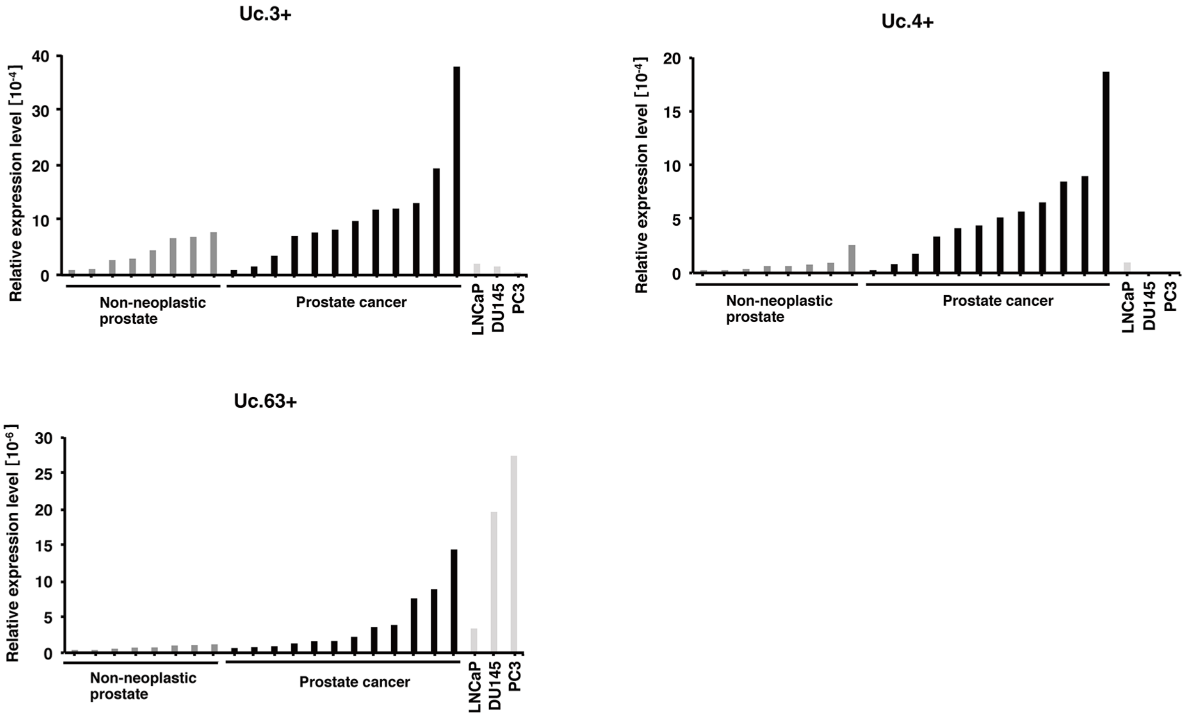

Supplementary Figure 2: The expression of Uc.63+ in PC cell lines was elevated compared with that in Uc.3+ and Uc.4+. Results of qRT-PCR for the expression of Uc.3+, Uc.4+, and Uc.63+ in non-neoplastic prostate tissues, prostate cancer tissues, and prostate cancer cell lines (LNCaP, DU145 and PC3).

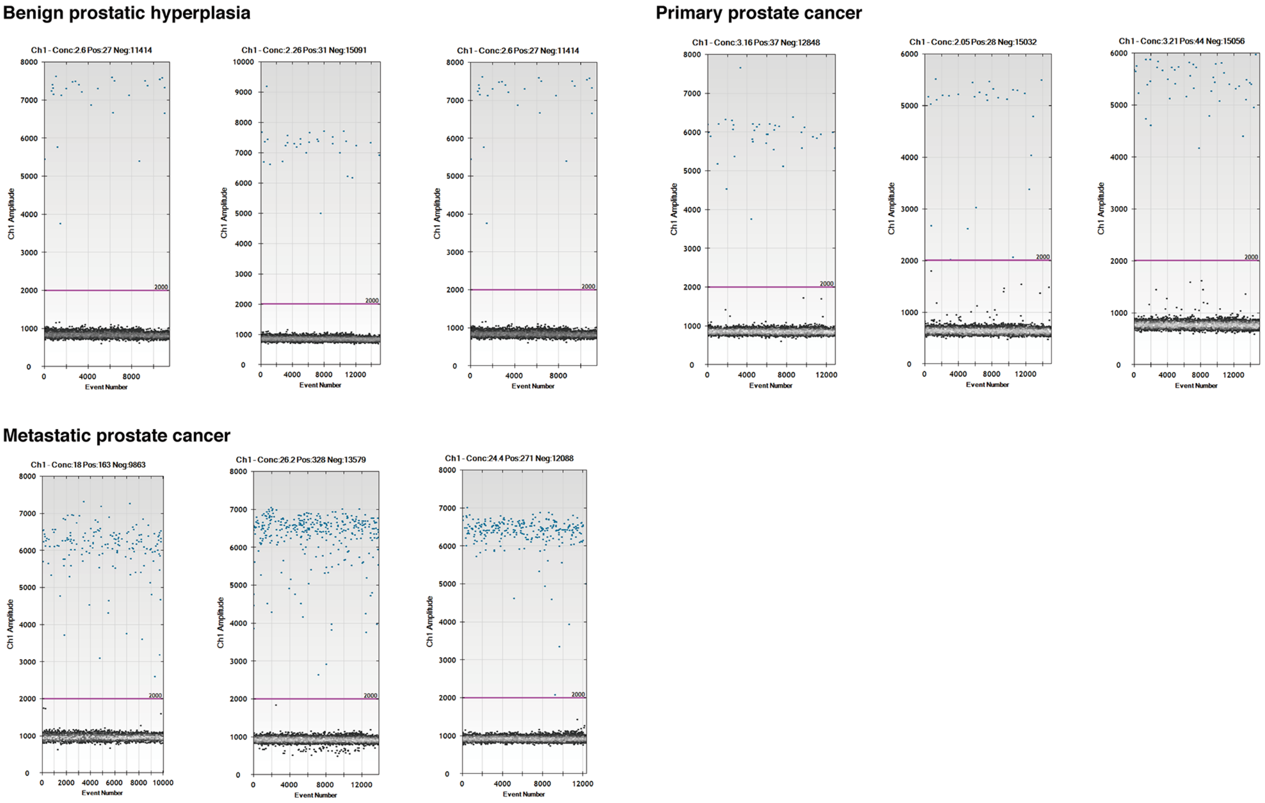

Supplementary Figure 3: Representative droplet digital PCR images. Results of droplet digital PCR for the expression of Uc.63+ in serum from patients with benign prostatic hyperplasia, primary prostate cancer, and metastatic prostate cancer.

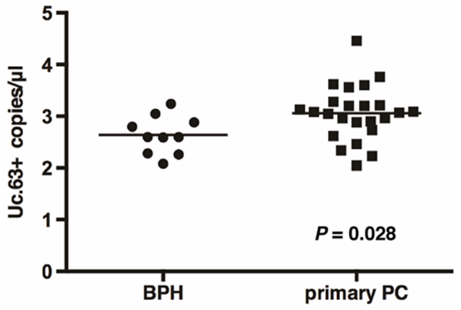

Supplementary Figure 4: The expression of Uc.63+ was elevated in the serum from primary PC compared with that in serum from BPH. Droplet digital PCR analysis for the expression of Uc.63+ in the serum of patients with benign prostatic hyperplasia (BPH) and primary prostate cancer (PC). Statistical differences were evaluated with the Mann-Whitney *U*-test.

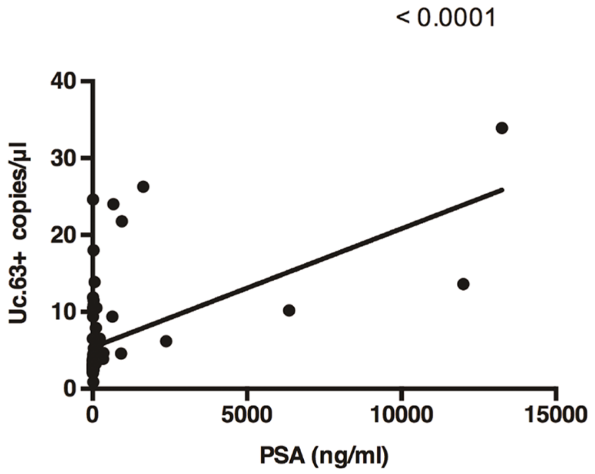

Supplementary Figure 5: The serum level of Uc.63+ was associated with PSA concentration. Correlation between the serum level of Uc.63+ and PSA concentration in the serum from primary prostate cancer and metastatic prostate cancer. *P* values are indicated.

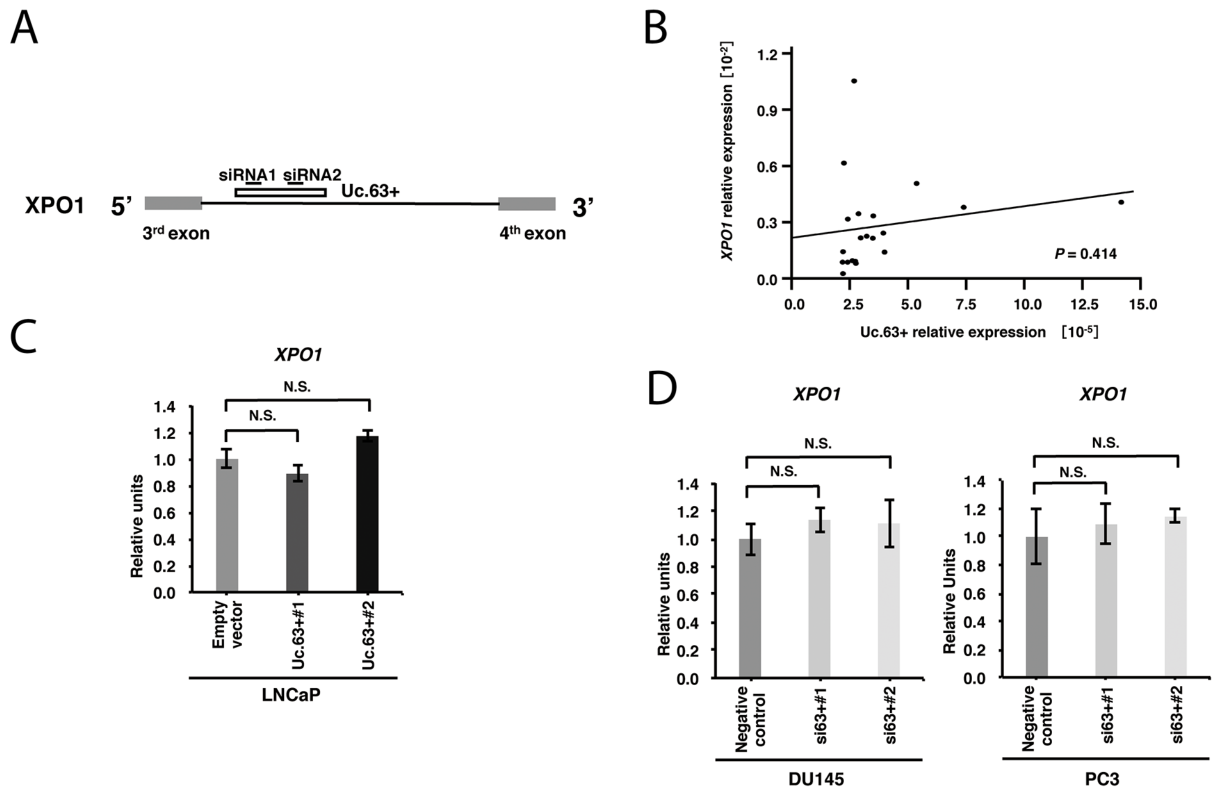

Supplementary Figure 6: Uc.63+ and XPO1 were independently regulated. (A) Schematic representation of intronic location of Uc.63+ within the *XPO1* gene (dark grey boxes: exons of *XPO1*, light gray box: Uc.63+). **(B)** Correlation between Uc.63+ and *XPO1* in prostate cancer tissues. Spearman correlation coefficient and *P* values are indicated. **(C, D)** qRT-PCR analysis of *XPO1* in LNCaP cells transfected with Uc.63+ expression vector or empty vector and in DU145 and PC3 cells transfected with negative control or two different siRNAs. Bars and error bars are the mean and S.D., respectively, of 3 independent experiments. N.S. (not significant).

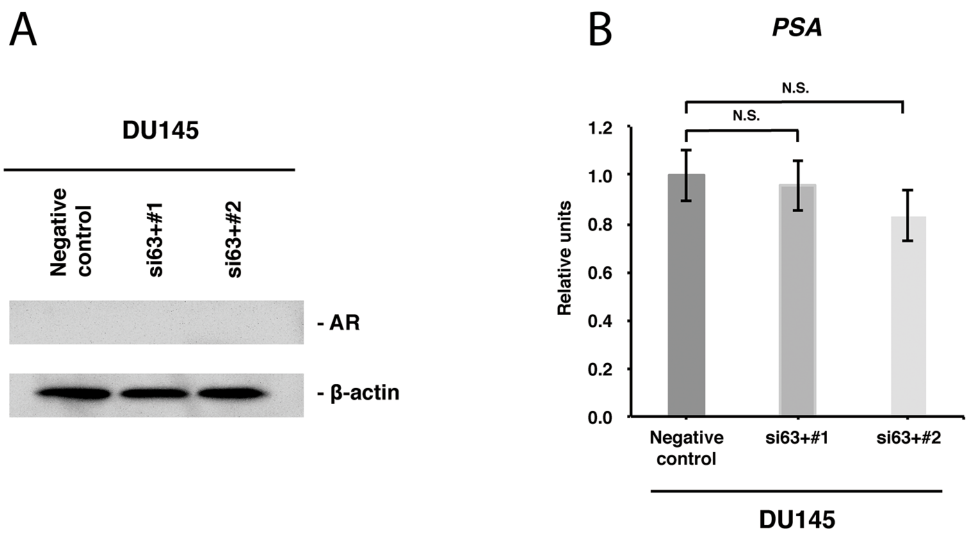

Supplementary Figure 7: Knockdown of Uc.63+ did not affect the expression of AR and *PSA* in DU145 cells. (A) Western blot analysis of AR and β-actin in DU145 cells transfected with negative control or two different siRNAs. β-actin was used as a loading control. **(B)** qRT-PCR analysis of *PSA* in DU145 cells transfected with negative control or two different siRNAs. Bars and error bars are the mean and S.D., respectively, of 3 independent experiments. N.S. (not significant).

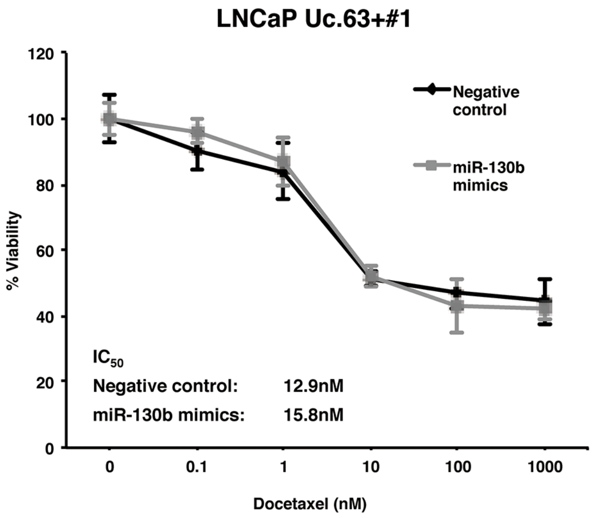

Supplementary Figure 8: The effect of miR-130b on docetaxel sensitivity in LNCaP transfected with Uc.63 (LNCaP Uc.63+#1) cells. Dose-dependent effect of docetaxel on the viability of LNCaP Uc.63+#1 cells transfected with miR-130b mimics or negative control.

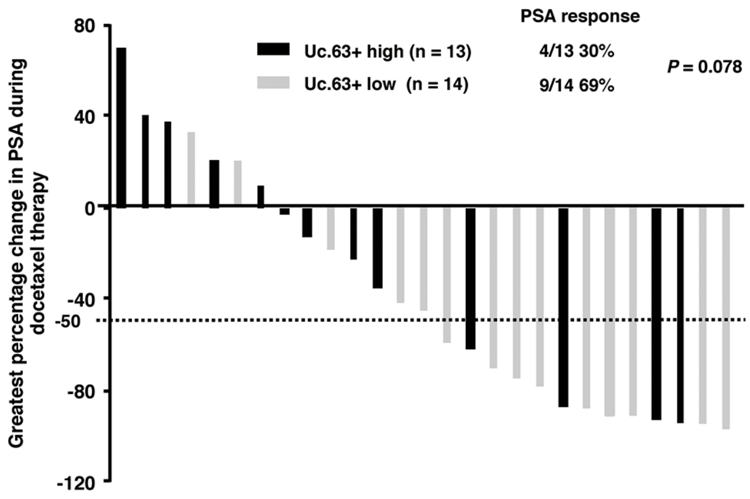

Supplementary Figure 9: Waterfall plot representing individual PSA responses. black box: Uc.63+ high, light gray box: Uc.63+ low. Dotted line marks a ≥50% reduction in PSA level from baseline, indicating a PSA response.

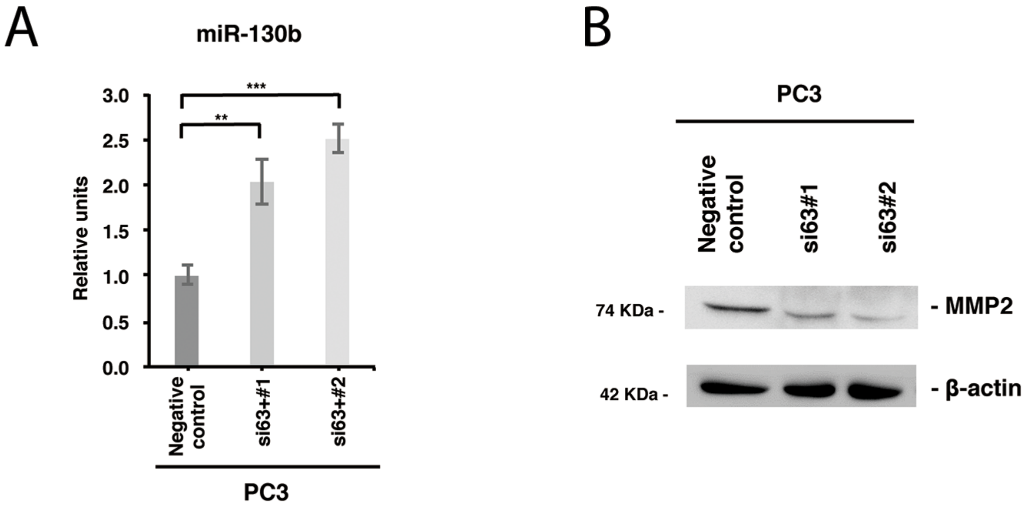

Supplementary Figure 10: The effect of knockdown of Uc.63+ on the expression of miR-130b and MMP2. (A) qRT-PCR analysis of miR-130b in PC3 cells transfected with negative control or two different siRNAs. Bars and error bars are the mean and S.D., respectively, of 3 independent experiments. $^{**}P<0.01$, $^{***}P<0.001$. **(B)** Western blot analysis of MMP2 and β-actin in PC3 cells transfected with negative control or two different siRNAs. β-actin was used as a loading control.

Supplementary Table 1: The clinicopathological characteristics of the patients with prostate cancer (PC)

Sample	PSA (ng/ml)	TNM stage	Gleason score
PC 01	10.5	T3aN0M0	3+4, ter.5
PC 02	494	T3bN0M1b	5+4
PC 03	7.3	T3aN0M0	3+4
PC 04	71.7	T3bN0M1b	4+4
PC 05	4.4	T2bN0M0	3+4
PC 06	8.6	T2aN0M0	4+3
PC 07	7.2	T2bN0M0	3+4
PC 08	12	T2bN0M0	4+4
PC 09	8.1	T2bN0M0	4+4
PC 10	8.4	T2bN0M0	3+3
PC 11	7.7	T3aN0M0	3+4
PC 12	4.8	T2bN0M0	4+5
PC 13	4.4	T2bN0M0	3+3
PC 14	11.2	T2cN0M0	5+4
PC 15	22.5	T2bN0M0	4+3 ter.5
PC 16	6.4	T2bN0M0	4+4
PC 17	4.6	T2cN0M0	4+3
PC 18	18.1	T3bN0M0	4+5
PC 19	5.6	T2cN0M0	4+3
PC 20	10.4	T2cN0M0	4+5

Supplementary Table 2: The clinicopathological characteristics of the patients with benign hyperplasia prostate, primary prostate cancer and metastatic prostate cancer.

See Supplementary File 1

Supplementary Table 3: Primers sequences of T-UCRs for qRT-PCR

T-UCRs	Forward primer	Reverse primer
Uc.3+	TTTGATTTGCATAACCCAACC	CCAAGGGCTGGATCAATCT
Uc.4+	GCAATCTCCATTCCGAAAGA	CCTGGCATCGGTATTTATGG
Uc.20+A	CTGTTTGCTCTGCCATTTCA	AATCCGCCTGCAGATAATTG
Uc.22+	TTCTTTCAAGAGAAAAGGGTAGC	TTTGCCTTTTGTGACCTGTG
Uc.33+	TCATTCCCTGTCTCCATTCC	TGGGGCGTAACCATCTTTAG
Uc.34+	TGACCAATACTTTTTGAAATTGATG	CAACCATCAAACCTCCCAAC
Uc.47+	GGTGACCTCACACACACCAA	TGAGGCAGCAAGATGTCATT
Uc.63+	TTGCATAAAAGCCAAATGTCA	CTGTTTGCTTGCCTGGTAAA
Uc.89+	GTGAGTTGCGCATAGACCAA	CATGAATCTGCAGCATATGATAGTT
Uc.106+	CATTTGCAGCCTAATTGTGG	TGCAAATCATTGGTGGTTTC
Uc.106+A	TGCAAATCATTGGTGGTTTC	CATTTGCAGCCTAATTGTGG
Uc.153+A	CACACCAGGGTCTTATAAACTGC	TTTCTCCTTCCCCCTCATTT
Uc.158+	TTTGTAATGAGAGCGGCTGA	TGCACGTTTCTCTTTGCATT
Uc.215+	TCAATGGACCGAGTGAAACA	AGGGCTCTGAAATTTGCTCA
Uc.217+A	CTGTCCGCTCCGTAGATTTT	CAGGTGCGAGGATAGCTACA
Uc.248+	GGGATCCCATTATGTGCTTG	GATGGGAATGTTTTCCATATCC
Uc.308+A	CACCATTCGGGCAGATTTAT	CTCAGAGATCGGCAGGAGTC
Uc.356+A	TTAATAACAGGCAGCGAGCA	TTGTGCATGCCTAGTGTTTTG
Uc.362+A	ACAAGGCAATCACACAACCA	GGGTAGTGCAAACTGGGAAA
Uc.363+A	CGAATAGCCAGAAGGTGAGTG	AAGCTGATTGGAGTGGAGGA
Uc.374+A	TGTCTTACAGGCCTTAATGTGC	GGATCTGATATGGCTTGAGCA
Uc.382+A	TGGCAACCAGACCCAAATAC	AAGGTCTTCCCATCCCTGTT
Uc.404+	ACAAATTGATTGCAAAAGAGC	TGCTGGCTTGATCAGATGTT
Uc.473+A	GCCCCCAAATACCTTCATCT	TGCAGTCATGCTGTTTTTGA
Uc.477+	GGCGACTACAAGACCACCAT	GATCACCTTGTCGGGATGTC
Uc.483+A	CTTCTGTGGCTGCGATTACA	TGAGCTGGGGATCTCATTTT

	Forward primer	Reverse primer
AR	GACGCTTCTACCAGCTCACC	GAAAGGATCTTGGGCACTTG
PSA	CACAGCCTGTTTCATCCTGA	AGGTCCATGACCTTCACAGC
XPO1	CAAATGTGAGAGCCTGCAAA	CGACCATCTGTGGATCATTG

Supplementary Table 4: Primers and probes sequences for ddPCR

Uc.63+ Forward	5'- AGCCAAATGTCATAGTGCATAA-3'
Uc.63+ Reverse	5'- CTGTTTGCTTGCCTGGTAAAT-3'
Uc.63+ FAM probe	5'FAM- ACCTGTTGCTTTCTTTCTGTTCCTCT -3'

Supplementary Table 5: The clinicopathological characteristics of the patients with metastatic castrate resistant prostate cancer (CRPC) treated with docetaxel

Sample	Gleason score	Baseline PSA (ng/ml)	Nadir PSA (ng/ml)	Greatest percentage change in PSA	PCWG3 PSA progression	ddPCR concentration (copies/µl)
CRPC 01	5+4	40	68	70	yes	21.8
CRPC 02	4+5	15	21	40	yes	18
CRPC 03	4+5	128	176	37	yes	13.9
CRPC 04	4+5	7	8.9	32	yes	4.6
CRPC 05	5+4	788	952	20	yes	53.9
CRPC 06	5+4	5	5.9	20	yes	4.9
CRPC 07	5+4	46	51.1	10	yes	5.2
CRPC 08	4+5	12	11.6	-3	yes	26.3
CRPC 09	4+5	284	246	-13	yes	24.4
CRPC 10	4+5	15	11.9	-18	yes	4.05
CRPC 11	4+4	26	19.8	-22	yes	24.6
CRPC 12	5+5	14	9.2	-36	yes	11.5
CRPC 13	3+4	39	22.8	-41	yes	4.6
CRPC 14	4+5	162	88.8	-45	yes	3.4
CRPC 15	4+4	59	24	-59	No	3.5
CRPC 16	5+4	73	28	-61	No	10.5
CRPC 17	5+4	39	11.5	-70	No	4.4
CRPC 18	4+4	137	34.7	-74	No	3.8
CRPC 19	3+3	70	15.1	-78	No	3.9
CRPC 20	5+3	2	0.2	-87	No	10.5
CRPC 21	5+3	20	2.4	-87	No	3.8
CRPC 22	3+5	28	2.6	-90	No	4.1
CRPC 23	5+5	156	13.9	-91	No	3.2
CRPC 24	5+5	7	0.4	-92	No	11.9
CRPC 25	4+4	4714	257	-94	No	10.2
CRPC 26	4+5	43	2.3	-94	No	5.2
CRPC 27	5+5	41	1.26	-96	No	3.2
