# Supplementary material for: Transcribed ultraconserved region Uc.63+ promotes resistance to docetaxel through regulation of androgen receptor signaling in prostate cancer
Source: Oncotarget. 2017 Oct 9;8(55):94259–70. doi: 10.18632/oncotarget.21688 (PMC5706872; doi:10.18632/oncotarget.21688)
Supplement: Supplementary file 2 [file oncotarget-08-94259-s002.docx]

**Supplementary Table 2**

The clinicopathological characteristics of the patients with benign hyperplasia prostate, primary prostate cancer and metastatic prostate cancer.

The clinicopathological characteristics of the patients with benign hyperplasia prostate.

| Number of cases | 10 |
| --- | --- |
| Median age (years) | 63 (51-78) |
| Median PSA concentration at diagnosis (ng/ml) | 6.2 (4.6-9.4) |
| Median ddPCR concentration (copies/μl) | 2.6 (2.0-3.2) |

The clinicopathological characteristics of the patients with primary prostate cancer.

| Number of cases | 24 |
| --- | --- |
| Median age (years) | 69 (54-76) |
| Median PSA concentration at diagnosis (ng/ml) | 8.7 (4.6-22.4) |
| Pathological T stage |  |
| pT2 | 18 (75%) |
| pT3 | 6 (25%) |
| Gleason score |  |
| 7 | 10 (41.6%) |
| 8 | 6 (25.0%) |
| 9 | 8 (33.3%) |
| Median ddPCR concentration (copies/μl) | 3.1 (2.0-4.7) |

The clinicopathological characteristics of the patients with metastatic prostate cancer..

| Number of cases | 45 |
| --- | --- |
| Median age (years) | 76 (50-84) |
| Median PSA concentration at diagnosis (ng/ml) | 50.2 (4.6-13500) |
| Gleason score |  |
| 7 | 3 (6.6%) |
| 8 | 10 (22.2%) |
| 9 | 28 (62.2%) |
| 10 | 4 (8.9%) |
| Metastatic sites |  |
| Lymph node | 42 |
| Bone | 39 |
| Median ddPCR concentration (copies/μl) | 5.2 (2.1-33.9) |
